# Utility of the HandScan in monitoring disease activity and prediction of clinical response in rheumatoid arthritis patients: an explorative study

**DOI:** 10.1093/rap/rkab004

**Published:** 2021-01-28

**Authors:** Maxime M A Verhoeven, Janneke Tekstra, Anne C A Marijnissen, Anna J L Meier, Antonius A A Westgeest, Floris P J G Lafeber, Johannes W G Jacobs, Jacob M van Laar, Paco M J Welsing

**Affiliations:** 1 Department of Rheumatology & Clinical Immunology, University Medical Center Utrecht, Utrecht; 2 Department of Rheumatology, Máxima MC, Eindhoven, The Netherlands

**Keywords:** rheumatoid arthritis, biological, DMARDs, optical spectral transmission, inflammation, disease activity

## Abstract

**Objectives:**

The aims were to determine the ability of the HandScan [assessing inflammation in hand and wrist joints using optical spectral transmission (OST)] to measure RA disease activity longitudinally, compared with DAS28, and to determine whether short-term (i.e. 1 month) changes in the OST score can predict treatment response at 3 or 6 months.

**Methods:**

Participants visited the outpatient clinic before the start of (additional) RA medication and 1, 3 and 6 months thereafter. Disease activity was monitored at each visit with the HandScan and DAS28 in parallel. A mixed effects model with DAS28 as the outcome variable with a random intercept at patient level, visit month and DAS28 one visit earlier was used to evaluate whether changes in the OST score are related to changes in DAS28. Binary logistic regression was used to test the predictive value of short-term changes in the OST score together with the baseline OST score for achievement of treatment response (EULAR or ACR criteria). All models were adjusted for RA stage (early or established).

**Results:**

In total, 64 RA patients were included. One unit change in OST score was found to be related to an average DAS28 change of 0.03 (95% CI: 0.01, 0.06, *P* = 0.03). When adding OST score as a variable in the longitudinal model, the ability of the model to estimate DAS28 (i.e. explained variance) increased by 2%, to 59%. Neither baseline OST score nor short-term change in OST score was predictive for treatment response at 3 or 6 months.

**Conclusion:**

A longitudinal association of OST score with DAS28 exists, although explained variance is low. The predictive ability of short-term changes in HandScan for treatment response is limited.

**Key messages**

- The HandScan measures inflammation in hand and wrist joints using optical spectral transmission.
- The HandScan (expressed as the optical spectral transmission score) is longitudinally related to disease activity as expressed by DAS28.
- The optical spectral transmission score should be combined with other parameters into a disease activity index for clinical practice.

## Introduction

The treatment of RA has improved significantly over the last decades owing to earlier and more intensive treatment, with swift adjustment of treatment if the target is not achieved [e.g. initiating biological DMARDs (bDMARDs)] [1, 2].

To treat RA patients effectively, it is important to focus on achieving and maintaining remission (treat-to-target principle), thereby preventing or restricting joint damage. Therefore, patients visit the outpatient clinic regularly to monitor disease activity (i.e. tight-control principle) [3, 4]. The DAS assessing 28 joints (DAS28) is widely used to evaluate disease activity in individual patients. Joint tenderness and swelling of 28 joints, together with an acute phase reactant (ESR or CRP) and a visual analogue scale for the patient’s experience of disease activity, are combined in the composite DAS28 measure. This method of evaluating disease activity has considerable inter- and intra-assessor variability, especially without formal training of assessors, and is time consuming and somewhat subjective [5].

The HandScan, based on the principle of optical spectral transmission (OST), is a new method that has been developed to measure RA inflammation in hand (i.e. MCP1–5, IP1 and PIP2–5) and wrist joints. The RA patient places both hands in the HandScan and, by using red/near-infrared light, the grade of inflammation is assessed per joint (i.e. individual joint score), in addition to providing a total score of all included joints (i.e. total OST score). A HandScan measurement can be performed at any location, if the device is available, within 5 min, without taking much time of a health-care professional [6]. More detailed information is provided in Supplementary Data S1, available at *Rheumatology Advances in Practice* online. In a cross-sectional study, the OST score as provided by the HandScan (range 0–66 = worst inflammation) was reproducible, and it was correlated (coefficient = 0.54) with the grade of inflammation of hand and wrist joints as assessed by ultrasonography [7]. The outcome of the HandScan was more sensitive in detecting subclinical disease activity (as determined by ultrasonography) than physical examination, and its assessment is less time consuming than that of DAS28 [8].

In addition, the HandScan might facilitate early detection of response to treatment, typically assessed at 3–6 months after the start of (added) therapy. This might be particularly relevant in (early) RA patients treated according to the tight-control principle, stepping up treatment to more intensive (biological) treatment modalities, such as TNF inhibitors (TNFi) [2].

All previous research with the HandScan was cross-sectional. However, in light of the treat-to-target principle, it is important to establish specifically whether changes in OST score are associated with changes in DAS28 (as reference standard) in individual RA patients (i.e. whether a longitudinal association of OST score with disease activity exists). Also, for optimal treat-to-target strategies, it would be valuable if the OST score could predict clinical response to treatment early after treatment initiation. Furthermore, during the last decades patient-reported outcomes have become of more interest as a measure for the impact of disease; therefore, the relationship of OST score to individual components of DAS28, functional disability and quality of life of patients is also of interest [9].

The aim of our explorative study was to determine the longitudinal association of the HandScan with DAS28 (i.e. whether changes OST score are related to changes in DAS28) in individual RA patients, which, if present and strong enough, would provide a rationale for its use as a disease activity monitoring instrument like DAS28. In addition, the longitudinal association of OST score with the swollen joint count (SJC), tender joint count (TJC), functional disability and quality of life of patients was determined.

Furthermore, the ability of short-term (i.e. baseline to 1 month) changes in OST score to predict clinical response to conventional synthetic DMARDs (csDMARDs) or TNFi treatment at 3 or 6 months was studied.

We hypothesized that a longitudinal association between OST score and DAS28 exists. Furthermore, we hypothesized that short-term changes in OST score can predict clinical response to treatment.

## Methods

This is an observational cohort study, among RA patients. The institutional review boards of the participating centres confirmed that the Medical Research Involving Human Subjects Act (WMO) was not applicable to this study, and all patients gave written informed consent.

Consecutive early and established RA patients visiting the outpatient clinic of participating centres, from 1 April 2017 to 31 May 2019, and satisfying the inclusion criteria were all eligible for inclusion. Inclusion criteria were meeting the 2010 ACR/EULAR criteria and age >18 years. Early RA patients were further required to be DMARD naïve and started DMARD therapy, usually a csDMARD such as MTX, according to the tight-controlled treat-to-target principle. Established RA patients started with or switched to another TNFi because of active disease, also in a tight-controlled manner, as additional therapy. Exclusion criteria for both cohorts were rheumatic autoimmune disease other than RA or a current inflammatory joint disease other than RA (e.g. gout). Other exclusion criteria were glucocorticoid use <6 weeks before baseline for early RA and previous use of the same TNFi (i.e. restarting treatment) for established RA.

All included patients visited the outpatient clinic immediately before starting their (additional) treatment (baseline) and 1, 3 and 6 months thereafter (i.e. tight controlled). In early RA patients, the csDMARD dose (typically MTX starting at 10 mg/week) was increased, if necessary, every month in steps of 5 mg, according to the treat-to-target principle. In established RA patients, the dose of the TNFi started was not modified during the study period of 6 months. Disease activity was measured at each visit, first with the HandScan and shortly afterwards with DAS28. The following baseline data were collected: age, gender, BMI, smoking status, alcohol use, RF status and anti-CCP status. DAS28 (and its components) and OST scores were collected at every visit, whereas the functional ability and quality of life were assessed at baseline and every 3 months thereafter, using the HAQ and EuroQol five dimensions questionnaire (EQ5D-5L), respectively.

### Statistical analysis

Baseline characteristics and treatment response were described for all patients and stratified by RA stage (early or established; csDMARD therapy or TNFi therapy). Data of early and established RA were combined to obtain a more adequate sample size. The effect of RA stage was taken into account in all model-based analyses (e.g. see explanation of the mixed effect models and the binary logistic regression models later in this subsection) [10]. Pearson or Spearman correlation coefficients, depending on the distribution of the data, of DAS28, SJC, TJC, HAQ and EQ5D-5L, with OST score were calculated for all patients, both concurrently and with time lags to explore the crude associations of OST score over time with other frequently used outcome measures. To determine whether changes in OST scores are related to changes in DAS28 in individual patients, an autoregressive mixed effects model with a random intercept at patient level was used [11]. The outcome variable was DAS28; independent variables were OST score, visit month, RA stage and DAS28 at previous visit (i.e. autoregressor). The same analyses were performed for SJC (square root transformed), TJC (square root transformed), HAQ and EQ5D-5L as respective outcome variables. It was also explored whether RA stage (early *vs* established; csDMARD *vs* TNFi) modified the association between OST score and the outcomes by adding the interaction term (e.g. OST score*RA stage). Binary logistic regression was used to test the predictive value of short-term (i.e. 1 month) change in OST score together with baseline OST score for the outcome EULAR good response (yes/no), and ACR50 response (yes/no) at 3 or 6 months. Baseline DAS28 and short-term (i.e. 1 month) change in DAS28 were also evaluated in a similar separate analysis for comparison with the former model. This analysis was also adjusted for RA stage (early *vs* established) because the initiated therapy differed (csDMARD *vs* TNFi), and it was tested whether RA stage modified the association between changes in OST score and outcome (i.e. adding the interaction term OST score*RA stage).

Owing to the exploratory nature of this study, no power calculation was performed. The statistical analyses were performed in SAS v.9.4 (SAS Institute, Inc; Cary, North Carolina, USA). All tests were two sided, and a *P*-value of ≤0.05 was considered statistically significant. Seven of 64 patients had missing information on DAS28 and/or OST score, but only at the 6 month visit. Given that mixed model analysis, using all longitudinally available data of the patients, is robust against sporadically missing data, imputation was deemed to have no additional value in this situation and was not performed [12].

## Results

In total, 64 RA patients were included: *n* = 32 with early RA (DMARD naïve, starting MTX and prednisone) and *n* = 32 with established RA (starting with first or consecutive TNFi as additional therapy). All early RA patients were treated according to EULAR guidelines and remained on MTX treatment during the study. Regarding established RA patients, 26 of 32 were bDMARD naïve and started treatment with a first TNFi, whereas the others started a consecutive TNFi. More detailed information about medication use is shown in Supplementary Table S1, available at *Rheumatology Advances in Practice* online. In early RA patients, no treatment failures during the 6 month follow-up were observed, whereas five patients in the established RA cohort discontinued TNFi therapy owing to insufficient effectiveness. Three of them switched after 3 months to another bDMARD. One of 32 established RA patients experienced an adverse event (not related to TNFi therapy) and stopped therapy.

**Fig. 1:**
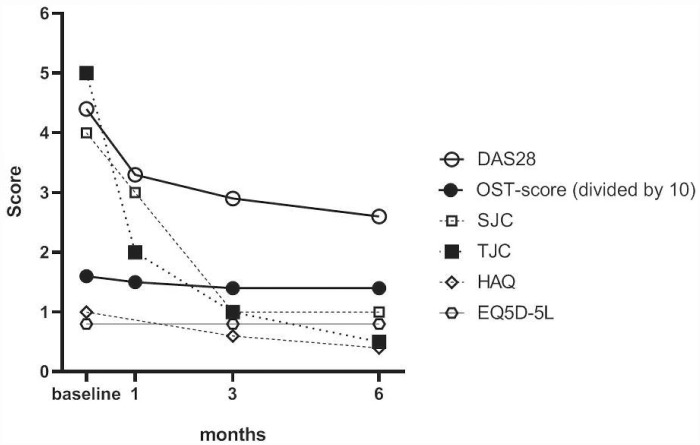
Disease activity measures, functional ability and quality of life over time *Means for DAS28 and OST score and medians for SJC, TJC, HAQ and EQ5D-5L. DAS28: DAS assessing 28 joints; EQ5D-5L: EuroQol five dimensions questionnaire; OST score: optical spectral transmission score; SJC: swollen joint count; TJC: tender joint count.

Table 1 provides an overview of the baseline characteristics and treatment response of all patients, and separately, per cohort. Overall, similar outcomes were observed for early and established RA, except for SJC28 and number of alcohol users, both at baseline, and response to treatment during the study period, except for changes in HAQ (see Table 1). The DAS28, OST score, SJC, TJC, HAQ and EQ5D-5L values over time are shown in Fig. 1.

**Table 1:** Baseline characteristics and treatment response

	All (*n* = 64)	Early (*n* = 32)	Established (*n* = 32)	*P*-value
**Baseline characteristics**				
Female, *n* (%)	42 (66)	22 (69)	20 (63)	0.60
Age (years), mean (s.d.)	57.6 (11.6)	55.8 (12.2)	59.6 (10.8)	0.26
BMI (kg/m²), mean (s.d.)	26.4 (4.9)	25.7 (5.1)	27.2 (4.5)	0.18
Duration of RA (years), median (IQR)	n.a	n.a.	8 (2–13)	n.a
Smoker, *n* (%)	13 (22)	7 (22)	6 (23)^a^	0.91
Alcohol user (≥1 unit/week), *n* (%)	36 (56)	22 (69)	14 (44)	0.04^*^
RF positivity, *n* (%)	48 (77)	23 (72)	25 (83)	0.51
Anti-CCP positivity, *n* (%)	50 (81)	29 (91)	21 (70)	0.11
DAS28, mean (s.d.)	4.4 (1.1)	4.5 (0.9)	4.3 (1.2)	0.39
SJC28, median (IQR)	4 (2–7)	6 (4–10)	3 (1–6)	0.01^*^
TJC28, median (IQR)	5 (2–8)	4.5 (3–7)	6 (2–11)	0.35
VAS global, median (IQR)	55 (38–70)	61 (42–75)	51 (30–63)	0.27
OST score, mean (s.d.)	15.6 (5.3)	16.8 (5.8)	14.4 (4.3)	0.10
HAQ score, median (IQR)	1.0 (0.6–1.4)	0.9 (0.5–1.4)	1.1 (1.0–1.7)	0.20
**Treatment response**				
ΔDAS28 month 3, mean (s.d.)	1.5 (1.3)	2.0 (1.0)	0.9 (1.3)	<0.01^*^
ΔDAS28 month 6, mean (s.d.)	1.9 (1.3)	2.5 (1.0)	1.1 (1.1)	<0.01^*^
ΔOST score month 3, mean (s.d.)	1.6 (4.6)	2.6 (4.7)	0.5 (4.3)	0.04
ΔOST score month 6, mean (s.d.)	1.7 (5.3)	2.6 (5.9)	0.7 (4.6)	0.10
EULAR good response month 3, *n* (%)	27 (42)	19 (59)	8 (25)	<0.01^*^
EULAR good response month 6, *n* (%)	34 (53)	28 (88)	6 (19)	<0.01^*^
ACR50 response month 3, *n* (%)	20 (31)	14 (44)	6 (19)	0.03^*^
ACR50 response month 6, *n* (%)	28 (44)	18 (56)	10 (31)	0.04^*^
ΔHAQ score month 3, median (IQR)	0.3 (0.0–0.8)	0.4 (0.1–0.8)	0.3 (0.0–0.8)	0.35
ΔHAQ score month 6, median (IQR)	0.5 (0.3–0.9)	0.6 (0.3–1.0)	0.4 (0.1–0.9)	0.24

a Six missing.

* Statistically significant.

All: early and established RA patients; Early: newly diagnosed RA patients starting conventional synthetic DMARD therapy; Established: established RA patients starting (first of new) TNF inhibitor as additional therapy. BMI= body mass index; CCP= cyclic citrullinated peptide; DAS28: DAS assessing 28 joints; HAQ= Health Assessment Questionnaire, range 0.-3=worst. *= statistically significant; IQR: interquartile range; OST: optical spectral transmission, range 0–66 (worst); RA= rheumatoid arthritis; RF= rheumatoid factor; SD=standard deviation; SJC28: swollen joint count assessing 28 joints; TJC28: tender joint count assessing 28 joints; VAS: visual analogue scale, range 0–100 (worst).

The concurrent (i.e. at the same time point) correlations between DAS28 and OST score and between SJC and OST score were moderate (correlation coefficients ranging from 0.18 to 0.39 and from 0.35 to 0.47, respectively) and statistically significant. Lower (often) non-statistically significant correlations of OST score were found with TJC, HAQ and EQ5D-5L (see Table 2). Non-concurrent correlations were also generally lower and often not statistically significant.

**Table 2:** Correlation coefficients between optical spectral transmission scores and DAS28/SJC28/TJC28/HAQ/EQ5D over time

	Optical spectral transmission score			
Measure	Baseline	Month 1	Month 3	Month 6
DAS28, baseline	0.31^*^	0.25^*^	0.27^*^	0.11
DAS28, month 1	0.17	0.18	0.26^*^	0.11
DAS28, month 3	0.26^*^	0.25^*^	0.39^*^	0.39^*^
DAS28, month 6	0.17	0.20	0.31^*^	0.34^*^
SJC, baseline	0.41^*^	0.49^*^	0.42^*^	0.39^*^
SJC, month 1	0.33^*^	0.47^*^	0.51^*^	0.50^*^
SJC, month 3	0.23	0.30^*^	0.38^*^	0.47^*^
SJC, month 6	0.30^*^	0.19	0.16	0.35^*^
TJC, baseline	0.00	−0.02	−0.02	−0.02
TJC, month 1	−0.05	−0.04	0.07	0.08
TJC, month 3	0.16	0.17	0.31^*^	0.39^*^
TJC, month 6	0.07	0.16	0.29^*^	0.46^*^
HAQ, baseline	0.08	–	0.20	0.01
HAQ, month 3	−0.01	–	0.10	0.10
HAQ, month 6	−0.23	–	0.08	0.09
EQ5D, baseline	−0.05	–	−0.21	−0.26
EQ5D, month 3	0.08	–	−0.15	−0.21
EQ5D, month 6	−0.05	–	−0.14	−0.35^*^

Outcomes are based on all patients (early and established, *n* = 64).

* Statistically significant.

DAS28: DAS assessing 28 joints; EQ5D: EuroQoL-5D; HAQ = health assessment questionnaire; OST: optical spectral transmission; SJC28: swollen joint count; TJC: tender joint count.

**Fig. 2:**
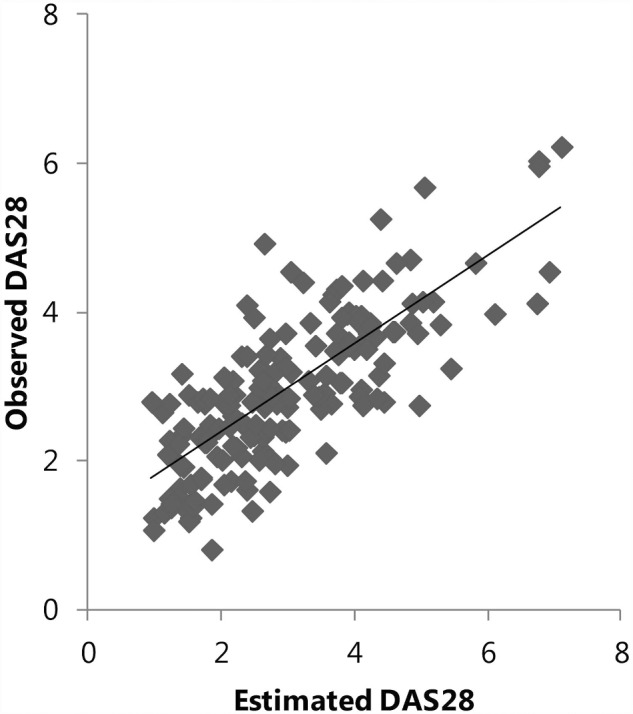
Observed DAS28 *vs* estimated DAS28 (using full model with optical spectral transmission score) DAS28: DAS assessing 28 joints; predicted DAS28: DAS28 as estimated by the model, with optical spectral transmission score, visit month and DAS28 at the previous visit as variables.

The longitudinal analysis showed that one unit change in OST score was associated with a change in DAS28 of, on average, 0.03 units (95% CI: 0.01, 0.06). Using standardized values, this could be interpreted as a change of one s.d. unit in OST score being related to a change in DAS28 of, on average, 0.13 s.d. unit (95% CI: 0.03, 0.23). Hence, changes in DAS28 value can, to some extent, be estimated from changes in the OST score. This association was not modified by RA stage (*P* = 0.96 for the interaction term). When adding OST score to the model with only the previous DAS28 (autoregressor) and visit, the ability of the model to estimate DAS28 over time (i.e. explained variance) increased, by 2%, to 59% (Fig. 2). Changes in SJC and TJC of one s.d. unit were, on average, related to changes of 0.18 (95% CI: 0.05, 0.31) and 0.16 (95% CI: 0.05, 0.25) s.d. units of OST score, respectively. The explained variance increased by 4 and 3%, respectively (to 32 and 43%, respectively) when adding the OST score to the models. The association with SJC (but not TJC, *P* = 0.52 for the interaction term) was found to be modified by RA stage (*P* = 0.03 for the interaction term). Stratified analyses showed that one s.d. unit of OST score change was, on average, related to 0.08 (95% CI: −0.08, 0.14) and 0.37 (95% CI: 0.15, 0.59) s.d. units in SJC, respectively, for early and established RA. No association of OST score with HAQ nor EQ5D-5L was found (results not shown).

Baseline OST score [odds ratio (OR) 0.93, 95% CI: 0.83, 1.04; standardized OR 0.67, 95% CI: 0.37, 1.22] and the short-term change in OST score (OR 1.04, 95% CI: 0.90, 1.19; standardized OR 1.18, 95% CI: 0.65, 2.13) were both not statistically significant predictors for EULAR response at 3 months. Baseline DAS28 was not a significant predictor (OR 0.76, 95% CI: 0.41, 1.41; standardized OR 0.74, 95% CI: 0.38, 1.44), whereas short-term change in DAS28 was a significant predictor (OR 4.47, 95% CI: 1.73, 11.58; standardized OR 3.96, 95% CI: 1.65, 9.52). Results for EULAR response at 6 months were in line with the above (see Table 3). For ACR50 response at 3 months, none of the variables were significant predictors. Short-term change in DAS28 (OR 3.92, 95% CI: 1.57, 9.28; standardized OR 3.69, 95% CI: 1.65, 9.52) was a significant predictor for ACR50 response at 6 months (see Table 3). In all analyses, the association of the short-term change in OST score with treatment response was not modified by RA stage as tested in the models (*P* = 0.44/*P* = 0.22 and *P* = 0.20/*P* = 0.30 for ACR50 and EULAR good response at 3/6 months, respectively).

**Table 3:** Odds ratios for predictive ability of variables on treatment response

Parameter	OR (95% CI) multivariable	OR (95% CI) multivariable
**EULAR good response at month 3**		
Baseline OST score	0.93 (0.83, 1.04)	
ΔOST score at month 1	1.04 (0.90, 1.19)	
Baseline DAS28		0.76 (0.41, 1.41)
ΔDAS28 at month 1		4.47 (1.73, 11.58)^*^
RA stage	0.20 (0.07, 0.62)^*^	0.41 (0.12, 1.37)
**EULAR good response at month 6**		
Baseline OST score	0.95 (0.93, 1.10)	
ΔOST score at month 1	1.07 (0.89, 1.27)	
Baseline DAS28		0.71 (0.34, 1.49)
ΔDAS28 at month 1		3.05 (1.05, 8.90)^*^
RA stage	0.03 (0.01, 0.13)^*^	0.04 (0.01, 0.19)
**ACR50 response at month 3**		
Baseline OST score	0.93 (0.82, 1.05)	
ΔOST score at month 1	1.10 (0.94, 1.31)	
Baseline DAS28		0.96 (0.55, 1.78)
ΔDAS28 at month 1		1.77 (0.81, 3.88)
RA stage	0.22 (0.06, 0.75)^*^	0.35 (0.10, 1.22)
**ACR50 response at month 6**		
Baseline OST score	0.94 (0.84, 1.05)	
ΔOST score at month 1	1.00 (0.88, 1.14)	
Baseline DAS28		0.83 (0.47, 1.49)
ΔDAS28 at month 1		3.92 (1.57, 9.28)^*^
RA stage	0.27 (0.09, 0.82)^*^	0.58 (0.18, 1.87)

* Statistically significant (OR 95% CI not including 1).

DAS28: DAS assessing 28 joints; Δ: change; OR: odds ratio, OST: optical spectral transmission; RA stage: early (starting conventional synthetic DMARD therapy) is reference.

## Discussion

In this first longitudinal study of the HandScan, the concurrent correlations of HandScan (expressed as OST score) and DAS28 were, in general, low to moderate, consistent with data of a previous cross-sectional study [8]. Although we established a longitudinal association of the HandScan with DAS28, which would be a prerequisite for using such an instrument for monitoring disease activity over time, the added value (explained variance) was low. This limits the use of the HandScan as a comprehensive instrument for monitoring disease activity in individual patients.

A plausible explanation for the low ability to estimate DAS28 with OST scores might be that, in addition to the number of tender and swollen joints (from 28), an acute phase reactant (i.e. ESR) and a visual analogue scale expressing the patients’ assessment of disease activity are part of DAS28 [13], whereas the OST score measures only RA inflammation of the hand and wrist joints (with a maximum of 22 joints). Therefore, we also evaluated components of DAS28 separately. The association with TJC and, especially, SJC was (somewhat) stronger than with DAS28, as apparent from the higher standardized regression coefficients and increase in explained variance by adding OST score to the longitudinal model.

We could not establish a predictive association of baseline OST score or short-term changes in OST score with later response to treatment. This lack of predictive ability might also be attributable, in part, to the fact that OST scores only reflect joint inflammation in a limited set of joints, and response criteria are based on composite scores [14].

As shown in Supplementary Table S1, available at *Rheumatology Advances in Practice* online, glucocorticoid therapy was used in early and established RA. Glucocorticoid therapy diminishes disease activity, but this will probably have been the case equally for DAS28 and the OST score. Therefore, we think that this has had no influence or only limited influence on the results of our main analysis (i.e. the longitudinal association between OST score and DAS28).

Given that inflammation of OA joints is generally considerably less than in RA joints, and the DIP joints (mostly affected in OA) are not assessed in the HandScan, we expect the influence of concomitant OA on OST score results to have been limited.

A limitation of this study is that the sample size is modest. The intention of the present study was to explore whether a longitudinal association of OST score with DAS28 existed, which is a prerequisite for using OST scores as a disease activity measurement in patients over time. Therefore, we aimed to include ≥30 early and 30 established RA patients. In the analyses, we combined early (*n* = 32) and established (*n* = 32) RA patients, correcting for RA stage. It turned out that RA stage did not influence the longitudinal association between OST score and other outcomes, except for SJC. A possible explanation might be the fact that in early RA patients the SJC was often zero at follow-up owing to the strict treat-to-target treatment approach, possibly obscuring small changes over time, whereas SJC was generally higher in established RA patients [15]. In addition, the predictive association between short-term changes in OST score and longer-term response was also not influenced by RA stage. Furthermore, one would expect that the type of treatment might influence the ability of OST scores to detect changes in disease activity, because bDMARDs are known to suppress tissue vascularity more rapidly [16, 17]. We tested whether the relationship between OST scores and DAS28(-based response) was different between early (i.e. csDMARD-treated) and established (i.e. bDMARD-treated) patients, but could not detect a significant effect. Of course, given that the effect of RA stage and treatment modality are intertwined in our study, this might have muddled this effect. In the bDMARD-treated group, patients could have started their next bDMARD, which could have diminished the potential change in joint vascularity (and thus the ability of the HandScan to detect it), because vascularity was already reduced by the previous bDMARD. It is known that even in patients with inadequate response to bDMARDs, progression of joint damage is inhibited [18], and thus probably also joint vascularity. Lastly, given that the HandScan measures only hand and wrist joints, it might be applicable mainly for the subset of RA patients with involvement of the hand joints.

This first study assessing the longitudinal association of the HandScan with disease activity measures relevant in monitoring treatment response warrants future research focusing on the development of a composite measure to assess disease activity where a joint count assessment (i.e. SJC and TJC) is replaced by OST scores. OST scores can be obtained without visiting a physician, because a HandScan measurement can be performed easily by a non-health-care professional, and at any location where the device can be placed; for example, in the outpatient waiting room. By implementing a disease activity index (including only variables that are assessed without visiting a physician, i.a. OST scores) into daily practice, the time of rheumatologists and/or nurse practitioners might be saved in busy outpatient clinics, because only those patients with active disease would require an additional visit to the rheumatologist or health-care professional for a more detailed assessment, including joint counts.

### Conclusion

A longitudinal association of OST score with DAS28 exists, although the relationship is weak. As such, in this setting the OST score as a single measuring instrument is insufficient to assess disease activity comprehensively in RA patients. However, combining the OST score with other (routinely used) disease activity parameters might result in an adequate composite disease activity measure.

## Supplementary Material

rkab004_Supplementary_Data
