# Supplementary material for: Utility of the HandScan in monitoring disease activity and prediction of clinical response in rheumatoid arthritis patients: an explorative study
Source: Rheumatol Adv Pract. 2021 Jan 28;5(1):rkab004. doi: 10.1093/rap/rkab004 (PMC7931797; doi:10.1093/rap/rkab004)
Supplement: rkab004_Supplementary_Data [file rkab004_supplementary_data.docx]

**Supplementary Data S1. Technical explanation of the HandScan.**

The HandScan is a (new) medical device, which is developed to assess disease activity in RA patients. The technology of the device is based on quantifying the inflammation related hemodynamic response to an applied stimulus.1 The speed and magnitude of blood pooling differs between inflamed and healthy tissue due to the vascular changes, which are associated with inflammation. The process of blood pooling is measured by diffuse optical transmission. Both hands of a patient are placed in the HandScan and illuminated with red/near-infrared light; the transmitted light is detected by a camera on the other side of the patient’s hands. Since blood is a strong absorber in the red/near-infrared region of the light spectrum, increases of pooling of blood as in arthritis will result in a decrease of the transmitted light, which is “translated” into a higher score. A non-healthcare professional (without medical background) can perform a HandScan measurement; within 5 minutes the OST-score (i.e., HandScan outcome) is produced, which provides a direct measure of inflammation of MCP1-5, IP1 and PIP2-5 and wrists bilaterally.

^1^= Meier AJ, Rensen WH, de Bokx PK, de Nijs RN. Potential of optical spectral transmission measurements for joint inflammation measurements in rheumatoid arthritis patients. J Biomed Opt. 2012;17:081420.

**Supplementary Table S1. Medication use**

|  | Early n= 32 | Established  n= 32 |
| --- | --- | --- |
| GC users, n (%)   - Dose in users mg/day, median (IQR) | 32 (100)  10 (10 – 10) | 10 (31)  5 (5 – 15) |
| MTX users, n (%)   - Dose in users mg/week, median (IQR) | 32 (100)  20 (15 – 25) | 19 (59)  20 (16 – 25) |
| Leflunomide users, n (%)   - Dose in users mg/day, median (IQR) | 0 (0) | 4 (13)  15 (10 – 20) |
| Sulfasalazine users, n (%)   - Dose in users mg/day, median (IQR) | 0 (0) | 2 (6)  1250 (1000 – 1500) |
| Hydroxychloroquine users, n(%)   - Dose in users mg/day, median (IQR) | 0 (0) | 6 (19)  300 (200 – 400) |
| TNFi users, n (%)   - Infliximab, n (%) - Adalimumab, n (%) - Etanercept, n (%) - Certolizumab pegol, n (%) - Golimumab, n (%) | 0 (0) | 32 (100)^1^   - 1 (3) - 10 (31) - 19 (60) - 1 (3) - 1 (3) |

^1^= 6 patients failed on TNFi. One of them experienced an adverse event (not related to TNFi therapy), but stopped therapy, and in 5 patients TNFi therapy was ineffective, 3 of them switched after 3 months to another bDMARD and the other 3 stopped their study participation.
